# Editorial: Exercise interventions: empowering individuals with neurological conditions

**DOI:** 10.3389/fresc.2026.1879942

**Published:** 2026-06-12

**Authors:** Adriano Zanardi da Silva, Audrin Said Vojciechowski, Manoela Ferreira

**Affiliations:** 1 Federal University of Parana, Curitiba, Brazil; 2 Universidade Positivo, Curitiba, Brazil; 3 The Canadian Donation and Transplantation Research Program, Edmonton, AB, Canada

**Keywords:** brain, exercise, neurology, neurorehabilitation, Parkinson's disease, physical activity, physical therapy, rehabilitation

Neurological conditions remain among the leading causes of long-term disability worldwide, frequently affecting mobility, cognition, independence, participation, and quality of life. Among populations such as people with stroke, Parkinson's disease, cerebral palsy, acquired brain injury, and peripheral nerve injuries, rehabilitation research has increasingly explored exercise not only as a strategy to improve physical conditioning but also as a broader therapeutic approach capable of influencing motor recovery, cognitive performance, confidence, participation, and overall well-being.

The studies included in this Research Topic highlight a valuable evolution in the field of neurorehabilitation. Collectively, they suggest that exercise should not be regarded as a generic intervention, but rather as a multidimensional rehabilitation strategy whose effectiveness relies on factors such as task specificity, intensity, repetition, feedback, contextual relevance, and patient engagement. Although the neurological populations and intervention approaches varied significantly across studies, several common themes emerged throughout this Topic.

Scalzo et al. examined the effects of physical activity interventions on upper limb function in individuals with chronic stroke. Although some positive effects were identified, the authors noted that improvements were generally small and not consistently clinically meaningful. These findings reinforce an important concept in neurorehabilitation: that general physical activity alone may not necessarily lead to recovery in specific functional areas. In many cases, meaningful motor improvements likely require interventions that are highly task-specific and aligned with the targeted deficits. This distinction remains particularly relevant in chronic neurological conditions, where gains may heavily depend on the specificity of practice and adequate therapeutic dosage.

The significance of intervention intensity and repetition was also evident in the longitudinal study by Opheim et al., which investigated repeated periods of intensive rehabilitation in children with cerebral palsy. The authors observed improvements in gross motor function across various GMFCS levels, with younger children demonstrating greater responsiveness to intervention. These findings support the current understanding of experience-dependent neuroplasticity and reinforce the potential of sustained, developmentally sensitive rehabilitation approaches. Instead of viewing rehabilitation as a short-term or isolated process, studies like Opheim et al. emphasize the value of repeated, progressive interventions over time.

Several contributions in this Research Topic also explored how technology and augmented feedback can improve rehabilitation outcomes. In their systematic review, Zou et al. examined virtual reality interventions for individuals with Parkinson's disease and reported potential benefits in areas such as balance, gait, cognition, and quality of life. Similarly, Hirsch and Mrachacz-Kersting investigated the effects of augmented feedback during gait training in children with cerebral palsy, highlighting the possible importance of visual, auditory, and tactile feedback in enhancing motor learning and gait performance.

Notably, however, these studies also underscore the complexity of technology-assisted rehabilitation. Methodological heterogeneity, variability in intervention protocols, and limited long-term evidence remain challenges within the literature. Furthermore, technology itself should not be regarded as the therapeutic mechanism. Its clinical value likely relies on its ability to enhance engagement, increase task salience, improve feedback delivery, and support meaningful, repetitive practice within functionally relevant contexts.

Another aspect emerging from this research topic concerns participation and ecological validity. Increasingly, neurorehabilitation research has shifted from focusing solely on impairment-based outcomes to understanding how interventions affect participation in real-world activities and long-term quality of life. In this context, MacDonald et al. demonstrated links between higher levels of moderate-to-vigorous physical activity and improved health-related quality of life in individuals with acquired brain injury. These findings reinforce the importance of encouraging physically active lifestyles beyond supervised rehabilitation settings.

Similarly, Villeneuve et al. introduced a structured outdoor cycling program for individuals who have experienced a stroke, focusing on feasibility, confidence, goal achievement, and participation in meaningful leisure activities. In addition to the physical aspects of cycling, the intervention addressed factors such as environmental adaptability, self-confidence, independence, and social involvement. Notably, although significant gains were made in cycling-related confidence and personal goals, broader mobility outcomes remained largely unchanged, further supporting the idea that rehabilitation results tend to be highly specific to domains.

The influence of personalized and context-specific rehabilitation was further illustrated in the case report by Camerota et al., which described the integration of neurocognitive therapeutic exercise and focal vibratory stimulation following sciatic nerve injury. Although limited by its single-case design, the report highlights how sensory, cognitive, and motor aspects can interact during functional recovery and pain management. At the same time, the Perspective article by Opheim et al. examined post-stroke spastic paresis rehabilitation from a broader clinical and implementation perspective, emphasizing issues related to accessibility, therapy intensity, and customized care. Together, these contributions serve as a reminder that neurorehabilitation remains fundamentally person-centred and cannot be reduced to standardized protocols alone.

The contributions in this Research Topic show that exercise rehabilitation includes a variety of strategies. The effects of these strategies depend on factors like dose, specificity, feedback, engagement, and ecological transfer. Overall, these contributions support a broader perspective of neurorehabilitation that combines neuroscience, motor learning, technology, participation, and real-world function to enable meaningful recovery.

Future research should continue to develop personalized, ecologically valid rehabilitation models that translate therapeutic gains into lasting participation, independence, and quality of life across diverse neurological populations.

